# A pilot study on biaxial mechanical, collagen microstructural, and morphological characterizations of a resected human intracranial aneurysm tissue

**DOI:** 10.1038/s41598-021-82991-x

**Published:** 2021-02-10

**Authors:** Devin W. Laurence, Hannah Homburg, Feng Yan, Qinggong Tang, Kar-Ming Fung, Bradley N. Bohnstedt, Gerhard A. Holzapfel, Chung-Hao Lee

**Affiliations:** 1 Biomechanics and Biomaterials Design Laboratory (BBDL), School of Aerospace and Mechanical Engineering, The University of Oklahoma, 865 Asp Ave., Felgar Hall 219C, Norman, 73019 USA; 2 Department of Neurosurgery, The University of Oklahoma Health Sciences Center, Oklahoma City, 73104 USA; 3 Biophotonic Imaging Laboratory, Stephenson School of Biomedical Engineering, The University of Oklahoma, Norman, 73019 USA; 4 Department of Pathology, The University of Oklahoma Health Sciences Center, Oklahoma City, 73104 USA; 5 Stephenson Cancer Center, The University of Oklahoma Health Sciences Center, Oklahoma City, 73104 USA; 6 Department of Neurological Surgery, Indiana University School of Medicine, Indianapolis, IN 46202 USA; 7 Institute of Biomechanics, Graz University of Technology, 8010 Graz, Austria; 8 Department of Structural Engineering, Norwegian University of Science and Technology, 7491 Trondheim, Norway; 9 Institute for Biomedical Engineering, Science and Technology, The University of Oklahoma, Norman, OK 73019 USA

**Keywords:** Biomedical engineering, Tissues, Cerebrovascular disorders

## Abstract

Intracranial aneurysms (ICAs) are focal dilatations that imply a weakening of the brain artery. Incidental rupture of an ICA is increasingly responsible for significant mortality and morbidity in the American’s aging population. Previous studies have quantified the pressure-volume characteristics, uniaxial mechanical properties, and morphological features of human aneurysms. In this pilot study, *for the first time*, we comprehensively quantified the *mechanical*, *collagen fiber microstructural*, and *morphological* properties of one resected human posterior inferior cerebellar artery aneurysm. The tissue from the dome of a right posterior inferior cerebral aneurysm was first mechanically characterized using biaxial tension and stress relaxation tests. Then, the load-dependent collagen fiber architecture of the aneurysm tissue was quantified using an in-house polarized spatial frequency domain imaging system. Finally, optical coherence tomography and histological procedures were used to quantify the tissue’s microstructural morphology. Mechanically, the tissue was shown to exhibit hysteresis, a nonlinear stress-strain response, and material anisotropy. Moreover, the unloaded collagen fiber architecture of the tissue was predominantly aligned with the testing *Y*-direction and rotated towards the *X*-direction under increasing equibiaxial loading. Furthermore, our histological analysis showed a considerable damage to the morphological integrity of the tissue, including lack of elastin, intimal thickening, and calcium deposition. This new unified characterization framework can be extended to better understand the mechanics-microstructure interrelationship of aneurysm tissues at different time points of the formation or growth. Such specimen-specific information is anticipated to provide valuable insight that may improve our current understanding of aneurysm growth and rupture potential.

## Introduction

An intracranial aneurysm (ICA) is a dilation of the cerebral artery wall that arises due to genetic characteristics^1^, abnormal blood flow at bifurcations of the artery^2^, or alterations in the endothelial cell function^3^. This focal dilation of the artery will grow and remodel in response to the altered hemodynamics and may eventually rupture, leading to subarachnoid hemorrhage. A recent cohort study^4^ showed that 35% of ruptured ICAs caused death of the patients, and another 29% resulted in moderate-to-severe disability. Although relatively asymptomatic, ICAs can be detected by cranial imaging, such as digital subtraction angiography, computed tomographic angiography^5^, 3D rotational angiography^6^, multi-detector computed tomography^7^ and magnetic resonance angiography^8^. The two common clinical interventions include endovascular coil embolization and microsurgical clip ligation, which aim to mitigate blood flow into the aneurysm, and thus, prevent further growth of the ICA and reduce the rupture potential. However, endovascular coil embolization suffers from a low packing density of the aneurysm space and a high aneurysmal recurrence rate^9–12^, while surgical clip ligation carries high procedural risks associated with the required craniotomy^13,14^. A comprehensive understanding of the aneurysm tissue’s biomechanical properties is crucial to improve existing treatment methods and to develop new therapeutics and devices.

Biomechanical properties of ruptured and unruptured aneurysm tissues have been investigated in the literature^15–25^; however, these studies were largely limited to uniaxial or pressure-inflation mechanical characterizations. For example, Scott et al.^21^ quantified the pressure-volume relationship for artery segment specimens with and without aneurysms. They found that the aneurysm tissues were less extensible than the healthy counterparts, and that most of the changes in the tension-stretch curves between the two tissue groups were in the low-stress region, suggesting possible alterations in the elastin component of the tissue. Steiger et al.^23^ mechanically characterized tissues from both the aneurysm neck and the aneurysm dome in addition to control strips from the parent artery, in which their primary focuses were on the comparisons of failure stress and strain, defined as the yield stress and strain, of different tissues. They found that the failure strain was not significantly different between the aneurysm neck (or the aneurysm dome) and the arterial control. On the other hand, the failure stress was approximately 50% lower for the aneurysm dome compared to the aneurysm neck. These observations suggest that there are distinct microstructural changes, occurring near the aneurysm dome, which might be associated with wall thinning or the degradation of key constituents (e.g., elastin). More recently, Costalat et al.^16^ aimed to examine the differences in the mechanical properties between ruptured and unruptured ICA tissues. Their results showed that the unruptured aneurysms were stiffer than the ruptured ones, but there were no statistical differences in the wall thickness (0.17-0.68 vs 0.29-0.45 mm, respectively). Thus, aneurysm growth is likely a continuously-evolving process that degrades the structural integrity of the tissue until the aneurysm eventually ruptures. These three studies have made fundamental progress towards understanding the biomechanical properties of aneurysm tissues at different stages of aneurysm growth; however, the uniaxial and pressure-based loading may not be optimal, and planar biaxial loading may capture the physiological deformations more accurately.

In addition to quantifying the mechanical differences associated with the aneurysm tissues, researchers have attempted to understand the tissue’s morphology at different phases of the aneurysm growth process, by using standard histological methods^26,27^ or other alternative microscopy techniques such as two-photon microscopy^16,28^. For example, Frösen et al.^26^ classified 66 aneurysm dome tissues into four morphological groups based on qualitative histological assessments: Type A—endothelialized wall with linearly aligned smooth muscle cells; Type B—thickened wall with disorganized smooth muscle cells; Type C—hypocellular wall with either myointimal hyperplasia or organizing thrombosis; Type D—an extremely thin thrombosis-lined hypocellular wall. They believe that these morphological groups follow the growth and remodeling of the aneurysm as it progresses towards rupture, which is supported by the increased rupture incidence associated with each group: 42% for Type A, 55% for Type B, 64% for Type C, and 100% for Type D. Moreover, Hasegawa et al.^27^ quantified the morphological changes of the aneurysm tissue after treatment with clip blades. Specifically, the treated tissues had a thickened, fibrous intima layer that was infiltrated by lymphocytes and a negligible amount of elastin fibers. On the other hand, Signorelli et al.^29^ used two-photon microscopy to qualitatively show a disorganized collagen fiber architecture near the aneurysm rupture zone. Furthermore, Cebral et al.^28^ used multi-photon microscopy to quantify the recruitment of the collagen fibers in unruptured aneurysms in response to the applied uniaxial loading. These studies have shown the complex changes in the constituent morphology and collagen fiber architecture associated with the aneurysm growth and rupture processes. However, recent advances have resulted in new methods, such as optical coherence tomography (OCT) or polarized spatial frequency imaging (pSFDI), which allow for non-destructive quantifications of the microstructure that may be useful to the biomechanical understanding of aneurysms. For example, the OCT data, as verified by standard histology, can demonstrate the localized microstructural damage to the collagen fiber architecture, which could then be paired with the pSFDI-quantified load-dependent collagen fiber architecture to comprehend how those localized changes would alter the tissue’s microstructure-mechanics interrelationship.

Thus, the objective of this study is to utilize a unified scheme to quantify the mechanical properties, collagen fiber architecture, and microstructural morphology of one resected human intracranial aneurysm tissue. This is accomplished by performing an extensive biaxial mechanical characterization of the aneurysm tissue to understand the tissue’s nonlinearity, anisotropy, and direction-coupling behaviors. The biaxial mechanical data is then used to evaluate the applicability of three common constitutive models. Next, *load-dependent* polarized spatial frequency domain imaging is performed to examine how the changes in the collagen fiber architecture of the aneurysm tissue in response to the mechanical loads. Finally, optical coherence tomography and standard histology are used to observe the aneurysm’s morphology and to establish connections to the bulk tissue’s mechanical properties.

## Methods

### Patient information and human aneurysm tissue acquisition

**Figure 1:**
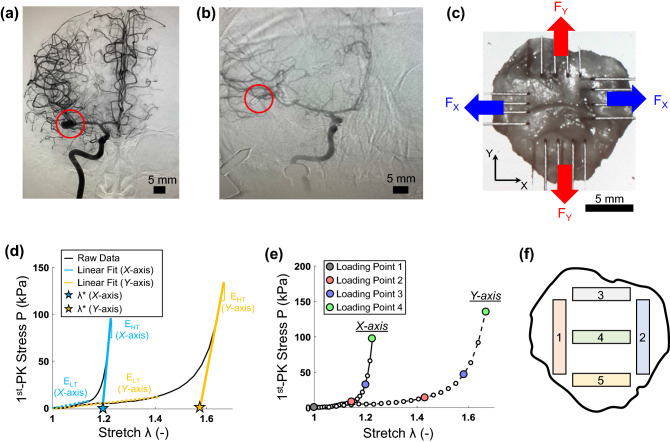
(**a**) Intraoperative angiogram showing the patient’s aneurysm prior to surgical clipping and (**b**) after operation. (**c**) Resected aneurysm tissue mounted to the CellScale BioTester. (**d**) Linear fits for the low-tensile modulus ($E_{LT}$) and high-tensile modulus ($E_{HT}$) of Protocol 1 ($F_X:F_Y=1:1$). Linear fits of the high-tensile modulus were used to determine $\lambda ^*$. (**e**) Definition of four loading points for the load-dependent pSFDI analysis *Loading Point* 1—unloaded; *Loading Point* 2—prior to the modulus transition; *Loading Point* 3—after the modulus transition; *Loading Point* 4—peak loading). (**f**) Location of the five tissue strips used for histological analysis.

A 49-year-old female with a history of migraines and smoking underwent a non-contrast head computed tomography (CT) that demonstrated a subarachnoid hemorrhage. Subsequently, she underwent CT angiography, which showed a right posterior inferior cerebellar aneurysm (Fig. 1a). In addition, a 3D rendering of the aneurysm was performed, and the 3D rotational angiography was reconstructed using a Syngo system (Siemens Healthineers, Malvern, PA, USA) for further analyses by the neurosurgeon. Subsequently, 2D digital subtraction radiographs were carried out at 3 frames per second, with a flow rate of 4 mL/sec, for a total of 7 mL of Isovue 300. This study was approved by the University of Oklahoma Health Sciences Center (OUHSC) (IRB#10039), and performed in accordance with the National Institutes of Health (NIH) guidelines on human subjects research and the OUHSC Institutional Review Board’s compliance policies. Informed consent form was obtained from the patient prior to the surgery.

Standard microsurgical dissection was performed to isolate the aneurysm. Clips were placed at the neck of the aneurysm, and micro-Doppler was used to confirm patency of the parent and branch vessels and lack of patency of the aneurysm. Aneurysm occlusion was next confirmed with intraoperative digital subtraction angiography (Fig. 1b). After confirming appropriate clipping, micro-scissors were used to incise and remove the aneurysm dome. The tissue was immediately placed in sterile saline, transported to the Biomechanics and Biomaterials Design Laboratory, and briefly frozen for storage until subsequent mechanical, collagen fiber microstructural, and morphological characterizations.

### Mechanical characterization

At the time of testing, the tissue was thawed, and thickness was determined by averaging three measurements acquired using a caliper (Westward 1AAU4, 0.01mm resolution). The tissue specimen was then mounted to a commercial BioTester system (CellScale, Canada) using four sets of four-tined Biorakes to create an effective testing region of $6.5 \times 6.5\ \hbox {mm}$ (Fig. 1c).

For mechanical characterization, the aneurysm tissue was submerged in a bath of $37\ ^{\circ }\hbox {C}$ phosphate-buffered saline that emulates the physiological blood condition, underwent 8 cycles of preconditioning to a target force of 900 mN, and then cyclically loaded/unloaded at the strain rates approximating the force-controlled loading to the target force ($F_{\mathrm{peak}}=900$ mN), considering various biaxial tension protocols (Table 1), to capture possible physiological deformations and mechanical coupling between the two testing directions. Load cell readings and tine-to-tine displacements were recorded at a rate of 1 Hz to calculate tissue stresses and stretches. Following the biaxial tensile characterizations, the tissue was deformed to the peak equibiaxial loading configuration and maintained for 15 min to characterize the tissue stress relaxation behavior.

**Table 1:** Experimental setups and quantified mechanics-related parameters for each of the seven biaxial tension protocols, showing loading ratio ($F_X:F_Y$), strain rate (%/s), portion of cycle, low-tensile modulus ($E_{LT}$), high-tensile modulus ($E_{HT}$) and index of extensibility ($\lambda ^*$).

Protocol	Loading ratio	Strain rate (%/s)		Portion of cycle	$E_{LT}$ (kPa)		$E_{HT}$ (kPa)		$\lambda ^*$ (-)	
	($F_X:F_Y$)	*X*-Dir	*Y*-Dir		*X*-Dir	*Y*-Dir	*X*-Dir	*Y*-Dir	*X*-Dir	*Y*-Dir
1	1:1	0.98	2.90	Loading	35.7	22.5	3158.2	1481.7	1.20	1.58
				Unloading	26.4	18.6	4260.1	2052.3	1.20	1.60
2	0.75:1	0.77	2.83	Loading	36.2	23.8	3808.2	1795.4	1.17	1.61
				Unloading	26.8	20.4	5064.7	2545.4	1.17	1.64
3	0.5:1	0.58	2.95	Loading	41.5	25.5	3876.6	1853.9	1.13	1.64
				Unloading	19.1	17.1	5267.0	2622.3	1.13	1.67
4	0.25:1	0.17	2.89	Loading	63.4	22.0	8168.5	2313.4	1.04	1.69
				Unloading	32.4	16.8	10453.4	3236.9	1.04	1.71
5	1:0.75	1.20	3.07	Loading	37.1	26.8	2939.2	1321.3	1.21	1.55
				Unloading	25.0	21.2	3905.7	1763.9	1.22	1.58
6	1:0.5	1.73	3.32	Loading	40.5	28.7	2233.1	905.1	1.25	1.47
				Unloading	25.7	23.0	2802.0	1151.7	1.26	1.49
7	1:0.25	2.38	2.60	Loading	47.4	35.2	1802.9	506.0	1.29	1.30
				Unloading	37.2	35.7	2311.9	637.6	1.31	1.32

### Analysis of the biaxial mechanical data

The load cell readings in both tissue directions were used to calculate the first Piola-Kirchhoff stress tensor $\mathbf{P}$, i.e., [$\mathbf{P}$] = $\mathrm{diag}[P_{XX}, P_{YY}] = \mathrm{diag}[F_X, F_Y]/(L \, t)$, where $P_{XX}$ and $P_{YY}$ are the *X*- and *Y*-components of $\mathbf{P}$, respectively, *L* is the initial specimen edge length, *t* is the initial specimen thickness, and $F_X$ and $F_Y$ are the load cell readings in the *X*- and *Y*-directions, respectively. In addition, the small displacements $\mathrm{d}X$ and $\mathrm{d}Y$ were used to calculate the tissue stretches in both directions: $\lambda _X = (L+\mathrm{d}X)/L$ and $\lambda _Y = (L+\mathrm{d}Y)/L$.

The biaxial mechanical data from the tension and stress relaxation protocols were further analyzed to determine key mechanics-related parameters of the stress-stretch responses. First, we quantified the low-tensile modulus $E_{LT}$ and the high-tensile modulus $E_{HT}$ from the stress-stress data of each of the 7 biaxial tension protocols (Table 1). These moduli were defined, for a given loading protocol and each testing direction, as the slope of the two straight lines (Fig. 1d). Next, the index of extensibility $\lambda ^{*}$ was determined in each tissue direction as the *x*-intercept of extension of the high-tensile modulus $E_{HT}$ (Fig. 1d).

### Constitutive modeling

Three common constitutive models were used to model the biaxial responses of human aneurysm tissue. The first was the simplified two-dimensional strain-based model proposed by Tong and Fung^30^, i.e.,

1 $$\begin{aligned} W=\frac{c}{2}\left[ \exp \left( a_1E_{XX}^2+a_2E_{XX}^2+2a_3E_{XX}E_{YY}\right) -1\right] , \end{aligned}$$

where *W* is the strain-energy function, *c*, $a_1$, $a_2$, and $a_3$ are the model parameters, and $E_{XX}$ and $E_{YY}$ are the Green-Lagrange strains in the *X*- and *Y*-directions, respectively.

The other two models use the first invariant $I_1$ of the right Cauchy-Green tensor $\mathbf{C} = \mathbf{F} ^{\mathrm{T}}{} \mathbf{F}$, where $\mathbf{F}$ is the deformation gradient, and the pseudo-invariants $I_4$ and $I_6$ which are equal to the squares of the stretches in the directions $\mathbf{N}$ and $\mathbf{M}$ of the two predominant fiber families, respectively. Both pseudo-invariants arise directly from the anisotropy and contribute to the strain-energy function *W*. The classical model proposed by Holzapfel et al.^31^ (i.e., the HGO model) does not consider a dispersion of the collagen fibers. We adopted the form^32^

2 $$\begin{aligned} W=C_{10}\left( I_1-3\right) +\frac{k_1}{2k_2}\left\{ \exp \left[ k_2(I_{4,1}-1)^2\right] -1\right\} +\frac{k_3}{2k_4}\left\{ \exp \left[ k_4(I_{4,2}-1)^2\right] -1\right\} . \end{aligned}$$

Herein, $C_{10}$, $k_1$, $k_2$, $k_3$, and $k_4$ are the model parameters, and $I_1 = \mathrm{tr}{} \mathbf{C}$, $I_{4,1}=\mathbf{N} \cdot (\mathbf{C} {} \mathbf{N} )$, and $I_{4,2}=\mathbf{M} \cdot (\mathbf{C} {} \mathbf{M} )$, where $[\mathbf{N} ] = [\cos \theta _1, \sin \theta _1]^{\mathrm{T}}$ and $[\mathbf{M} ] = [\cos \theta _2, \sin \theta _2]^{\mathrm{T}}$ denote the directions of the two fiber families in matrix form. The other model proposed by Gasser et al.^33^ (i.e., the GOH model) has the form

3 $$\begin{aligned} W=C_{10}\left( I_1-3\right) +\frac{k_1}{2k_2}\sum _{i=1}^{2} \left( \exp \left\{ k_2\left[ \kappa (I_1-3)+(1-3\kappa )(I_{4,i}-1)\right] ^2\right\} -1\right) , \end{aligned}$$

which is similar to Eq. (2), but it has an additional parameter $\kappa \in [0,1/3]$ describing fiber dispersion. The incompressibility condition, i.e., $J = \mathrm{det}{} \mathbf{F} \equiv 1$, was here enforced by the condition $F_{33}=(F_{11}F_{22} -F_{12}F_{21})^{-1}$.

The constitutive model parameters for Eqs. (1)–(3) were determined by fitting the models to the biaxial mechanical data by minimizing the following error function $e_j$ via an in-house differential evolution optimization (DEO) algorithm^34^, i.e.

4 $$\begin{aligned} e_j=\frac{1}{n_{\mathrm{data}}}\left[ \sum _{i=1}^{n_{\mathrm{data}}} \left( P_{XX,i}^{\mathrm{exp}}-P_{XX,i}^{\mathrm{model}}\right) ^2+\left( P_{YY,i}^{\mathrm{exp}} -P_{YY,i}^{\mathrm{model}}\right) ^2\right] ^{\frac{1}{2}}, \qquad j=1,\ldots ,n_{\mathrm{pop}}. \end{aligned}$$

Herein, $e_j$ denotes the error for the $j{\mathrm{th}}$ set from the $n_{\mathrm{pop}}$ sets of material parameters, $n_{\mathrm{data}}$ is the number of experimental data, and the superscript denotes either the experimental data or the model prediction. The optimization procedure was repeated 10 times and the average parameters were used to model the aneurysm tissue mechanical response. Additionally, the residuals were calculated for each experimental data point to visualize the model performance throughout the loading protocols.

### Quantification of tissue’s collagen fiber architecture

Following the mechanical characterization, an in-house pSFDI device was used to quantify the load-dependent changes in the tissue’s collagen fiber architecture (CFA). Details of this system are described previously^35^. Briefly, a LED-driven, micromirror-based pattern projection system (Texas Instruments, Dallas, TX) emitted cyan light (490 nm wavelength) through a rotating linear polarizer (Thorlabs, Newton, NJ) onto the tissue specimen, which was then reflected back through the same polarizer and captured as an 8-bit grayscale image by a 5 megapixel CCD camera (Basler, Germany). This process was repeated with 5$^{\circ }$ increments of the rotating polarizer between 0$^{\circ }$ and 180$^{\circ }$ (37 total images). The images were then processed to determine the pixel-wise fiber orientation angle $\theta _{\mathrm{fiber}}$ and the degree of optical anisotropy (DOA) that is related to the alignment of collagen fibers. The DOA values can range from 0 to 1, with smaller DOAs (i.e., DOA $=0$) denoting a random fiber network and larger DOAs (i.e., DOA $=1$) signifying highly-aligned fibers. More information regarding the determination of these parameters from the experimental data can be found in Section 2.1 of Jett et al. ^35^.

For investigations of the load-dependent changes in the CFA, the pSFDI device was integrated with the BioTester. The data of the equibiaxial tension protocol were used to determine four loading points along the force-displacement curve (Fig. 1e): *Loading Point* 1—unloaded, *Loading Point* 2—prior to the modulus transition, *Loading Point* 3—after the modulus transition, and *Loading Point* 4—peak loading. The tissue was displaced to the specified size of each of the above 4 loading points, and the pSFDI was performed to quantify the corresponding CFA.

The predicted angle $\theta _{\mathrm{fiber}}$ and DOA at the four mechanical loading points were used to generate probability density histograms for the tissue’s region of interest (i.e., the region bounded by the tines of BioRakes), which were then compared using the median and interquartile range (IQR). The values for *Loading Point* 1 (i.e., unloaded) were considered as the baseline, whereas the relative percent changes were calculated for *Loading Points* 2-4. Similar comparisons were made for a 3$\times$3 smaller sub-regions to examine the regional variations in the quantified CFA; more details can be found in the online Supplementary Information (Fig. S1).

### Characterization of tissue-level morphology

An OCT system (VEG220C1—$16\ \upmu \hbox {m}$ axial resolution and $20\ \upmu \hbox {m}$ lateral resolution, Thorlabs, Newton, NJ) was then placed above the mounted tissue. A standard OCT imaging method was used to capture the 3D microstructure of the tissue. Briefly, a laser beam was projected from the OCT system and divided into a reference part and a sample part. The sample part of the laser beam reflected off the tissue specimen and interfered with the reference portion of the laser, which formed the interference fringes that were received by a balanced detector. Depth-resolved tomography can be achieved by performing fast Fourier transform of the interference fringes^36^. After imaging, the 3D OCT data were post-processed to visualize the collagenous medial layer using a weighted mean filter^37,38^ and a weighted mean binarization algorithm^39^. This was performed for all 1280 cross-sectional images of the OCT data to create a 3D intensity map of the collagenous medial layer and to visualize the microstructural damage of the aneurysm tissue.

### Histological analysis

Routine histological methods were next employed to evaluate the extracellular matrix constituents of the aneurysm tissue. Specifically, five strips from different locations of the human aneurysm tissue were fixed in formalin for 48 h and stored in ethanol until processing (Fig. 1f). Next, each tissue strip was paraffinized, sectioned at a thickness of $5\ \upmu \hbox {m}$ using a microtome, deparaffinized, dehydrated, and placed onto a glass slide. Five glass slides were procured for each of the five regions of the human aneurysm tissue (25 total slides), and then stained with Masson’s Trichrome (for collagen fibers), Alcian Blue (for glycosaminoglycans and proteoglycans), Verhoeff van-Gieson (VVG, for elastin fibers), Hematoxylin and Eosin (H&E, for cell cytoplasm and nuclei), and von Kossa (for calcium). Results from the selected five regions of the aneurysm tissue (Fig. 1f) were qualitatively evaluated to understand the morphological changes in the extracellular matrix, such as loss of elastin or thickening of the intima tissue layer—morphological indicators of a developed aneurysm reported by other researchers^26,27^.

## Results and discussion

### Biaxial mechanical response

#### General observations

**Figure 2:**
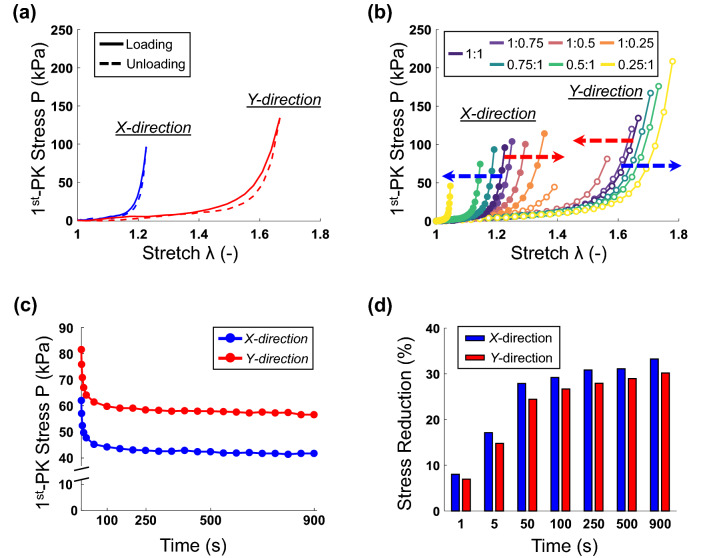
Biaxial mechanical results of (**a**) the loading and unloading cycles of the equibiaxial tension protocol ($F_X:F_Y=1:1$), (**b**) complete in-plane biaxial protocols, (**c**) stress relaxation protocol, and (**d**) percent stress reduction in stress relaxation.

The biaxial mechanical results are summarized in Fig. 2a,b and Table 1. Three key observations were made regarding the aneurysm tissues: (1) loading/unloading behavior (i.e., minimal hysteresis), (2) nonlinearity, and (3) material anisotropy and directional coupling. Similar properties have been displayed for other soft collagenous tissues, such as skin^40^, heart valve leaflets^41^ and arterial tissue^42^.

Firstly, Fig. 2a shows the difference between the loading and unloading curves for Protocol 1 (i.e., equibiaxial tension protocol, $F_X{:}F_Y=1{:}1$) of the aneurysm tissue. When comparing the unloading curve to the loading one, there was a decrease in the low-tensile modulus $E_{LT}$ and an increase in the high-tensile modulus $E_{HT}$ for both the *X*- and *Y*-testing directions, but the index of extensibility $\lambda ^*$ only changed (increased) in the *Y*-direction.

Secondly, Fig. 2a shows that the mechanical behavior of the aneurysm tissue was nonlinear with a soft toe region transitioning into a stiffer higher-stress region. This is quantitatively shown by a larger value of $E_{HT}$ compared to $E_{LT}$ (e.g., 3158 kPa vs 36 kPa for the *X*-direction of Protocol 1). The nonlinear mechanical response was consistent for both *X*- and *Y*-directions and throughout all loading protocols. Interestingly, both the low-tensile and the high-tensile moduli for the *X*-direction ($E_{LT}=36$–63 kPa and $E_{HT}=$1803–8169 kPa) were typically larger than those for the *Y*-direction ($E_{LT}=19$–35 kPa and $E_{HT}=506$–2313 kPa).

Thirdly, the mechanical response of the aneurysm tissue was anisotropic with a unique directional coupling (Fig. 2b). Quantitatively, the material anisotropy (with the *Y*-direction being more extensible) was demonstrated by the larger $\lambda ^*$ value in the *Y*-direction (1.30–1.60) compared to the *X*-direction (1.04–1.29) for all biaxial tension protocols. On the other hand, the directional coupling can be examined by means of the changes in $\lambda ^*$ for the non-equibiaxial loading protocols. Taking Protocol 4 (i.e., $F_X{:}F_Y=0.25{:}1$) as an example, the index of extensibility $\lambda ^*$ reduced in the direction of the reduced load (*X*-direction, 1.04 vs 1.20) and increased in the direction of the consistent load (*Y*-direction, 1.69 vs 1.58) as compared to Protocol 1. This trend was consistent for all the non-equibiaxial loading protocols (i.e., Protocols 2–7, see Table 1); however, there were larger changes in $\lambda ^*$ for the direction of the reduced load compared to the direction with the consistent load (1.7–21.0% vs 1.9–10.6%) and larger changes when the applied tension in the *Y*-direction was reduced (1.7–21.0% for the reduction in the *Y*-direction vs 2.9–14.6% for the reduction in the *X*-direction). This interesting directional coupling of the human aneurysm tissue is similar to the observations reported by May-Newman and Yin^43^ for the uniaxial and biaxial mechanical responses of porcine mitral valve leaflets.

Finally, the stress relaxation results (Fig. 2c) showed that there is a strong relaxation behavior of the tissue with the majority of relaxation occurring within the first 100 seconds. Beyond 100 seconds, the stress relaxation behavior reaches a plateau with a relatively smaller slope ($-0.004$ and $-0.006$ kPa/s in the *X*- and *Y*-directions, respectively) compared to the initial 5 seconds of the stress relaxation testing ($-2.126$ and $-2.411$ kPa/s in both directions). The *X*-direction of the tissue had a consistently larger percent reduction in the measured stress (Fig. 2d), with a 3% difference at the end of the stress relaxation testing (33.2% vs 30.2%). This unique stress relaxation behavior is likely caused by fiber re-orientation and fiber slippage within the collagen fiber architecture. These microstructural mechanisms are mitigated by proteoglycans (PGs) and/or glycosaminoglycans (GAGs). Fiber-fiber connections established by these proteins prevent the fiber-fiber slippage and, thus, limit the stress relaxation behaviors^44,45^. Therefore, it is possible that the specific human ICA tissue characterized in our study had a lower presence of PGs or GAGs in the ground susbstance, as indicated by the relatively large stress relaxation behaviors.

#### Comparisons with previous studies

Costalat et al.^16^ used uniaxial testing to classify meridionally-oriented (i.e., oriented along the arterial axial direction) ICA dome tissues ($n=15$) as having soft, intermediate, or stiff mechanical responses. The biaxial deformations in the present study were larger than the uniaxial deformations for all three classifications, with the *X*-direction being closer to the uniaxial deformations (0.25 vs 0.07-0.13) than the *Y*-direction (0.89 vs 0.07-0.13). Interestingly, the ICA tissue in the present study had smaller values for the low-tensile modulus in the *X*-direction (35.7 kPa) and the *Y*-direction (22.5 kPa) than all three tissue groups in Costalat et al. (371-1645 kPa), but had comparable high-tensile modulus values to the intermediate group in the *X*-direction (3158 kPa vs 3656 kPa) and to the soft group in the *Y*-direction (1482 kPa vs 1148 kPa).

Previous studies have also quantified the failure properties of aneurysm tissues. Robertson et al.^20^ showed that the average peak failure strain for aneurysm tissue strips from the meridional orientation ($n=8$) had a peak failure strain of 0.36. The peak strain of our aneurysm tissue in the *X*-direction (0.25) falls within this range whereas the *Y*-direction (0.89) does not, which further supports the idea that the *X*-direction of our tissue is approximately aligned near the meridional axis. On the other hand, Parshin et al.^19^ showed notably larger values for the peak failure strain of one posterior inferior cerebellar aneurysm sample compared to the average in Robertson et al. (2.86 vs 0.36), and the peak deformations in both directions of our aneurysm sample fall within this range. The large difference in the peak failure strains from these studies may suggest that Parshin et al. tested an aneurysm sample approximately oriented in the circumferential direction (i.e., orthogonal to the meridional direction).

Although the *in vivo* configuration of the tested ICA tissue is unknown, the above comparisons lead to the hypothesis that the tested *X*-direction of our aneurysm sample may be more aligned with the meridional direction of the artery. It is also likely that our aneurysm sample has a soft-to-intermediate mechanical response using the classification presented by Costalat et al.^16^. However, as outlined in **Study limitations and future extensions**, comparisons with the data of ICA tissues characterized using this technique are required to determine the accuracy of these statements.

### Constitutive modeling of the biaxial mechanical response

**Figure 3:**
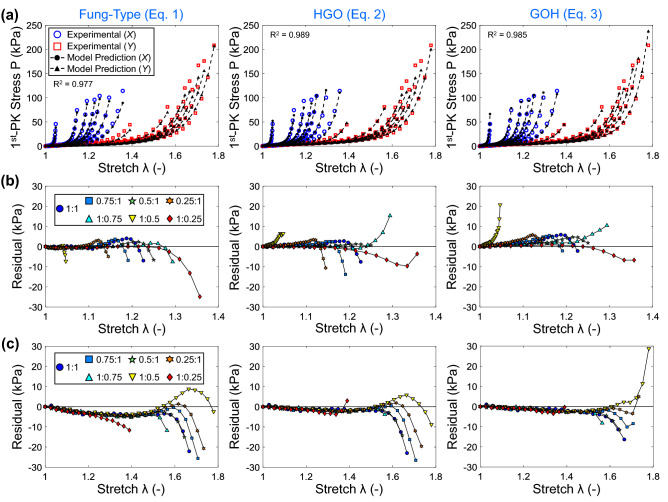
(**a**) Model fits to the experimental data for the three constitutive models. Residual values of the $1^{\mathrm{st}}$-PK stress for the deformation in the (**b**) *X*-direction, and (**c**) *Y*-direction.

The three constitutive models exhibited excellent fits to the biaxial mechanical data (Fig. 3a). The Holzapfel-Gasser-Ogden (HGO) model (Eq. (2)) appeared to have the best fit to the experimental data ($R^2=0.989$) compared to the Gasser-Ogden-Holzapfel (GOH) model (Eq. (2), $R^2=0.018$) and the Fung-type model (Eq. (1), $R^2=0.977$). Interestingly, both the HGO model and the GOH model predicted a similar orientation for the second fiber family ($30.0^{\circ }$ vs $28.6^{\circ }$) but a much different orientation for the first fiber family ($59.1^{\circ }$ vs $85.0^{\circ }$). The estimated model parameter information for the 10 optimization runs can be found in the online Supplementary Information (Table S1–S3). The plots of the first Piola-Kirchhoff ($1^{\mathrm{st}}$-PK) stress residual highlight the differences in the fitting performance of the three models in each loading protocol (Fig. 3b,c). The three models had moderate residual values throughout the low-tensile region, and the residuals become more exacerbated as the mechanical response transitions into the high-tensile region. Most importantly, these results highlight that despite being purely phenomenological, the Fung-type model can reasonably capture the biaxial mechanical behaviors of the intracranial aneurysm tissue. It would be ideal to use the HGO or GOH models in most modeling applications owing to their consideration of the collagen fiber architecture, their excellent performance and invariant-based three-dimensional formulations; however, the phenomenological Fung-type model can perform considerably well when information regarding the underlying collagen fiber architecture is scarce or is not critical for the simulation. Nevertheless, the three models presented herein appear to reasonably capture the biaxial mechanical responses of our human aneurysm tissue, but future extensions are warranted to thoroughly evaluate and establish constitutive model forms appropriate for modeling the nonlinear biaxial mechanical behaviors of human aneurysms.

### Collagen fiber architecture

#### General observations

**Figure 4:**
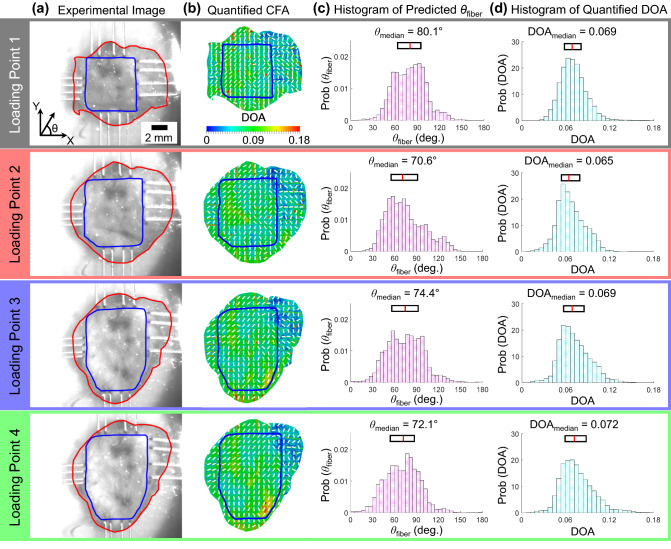
(**a**) Defined region of interest, (**b**) preferred collagen fiber orientation vector maps with a degree of optical anisotropy (DOA) color map, (**c**) histogram of the preferred fiber orientation, and (**d**) histogram of the DOA for the four loading points defined in the equibiaxial loading protocol (see Fig. 2d).

The pSFDI results provide insight into the function-structure relationship of the aneurysm tissue. For example, the unloaded tissue (i.e., *Loading Point* 1) had collagen fibers predominantly aligned close to the *Y*-direction ($\theta _{\mathrm{fiber}}=80.1^{\circ }$ and degree of optical anisotropy— DOA=0.069). As the tissue was equibiaxially loaded (Fig. 4), the median preferred fiber orientation angle $\theta _{\mathrm{fiber}}$ shifted away from the *Y*-direction ($-10.1$%) and the median DOA increased ($+5.1$%) at *Loading Point* 4 compared to the unloaded state. Thus, the collagen fiber architecture had load-dependent properties with the fibers becoming more aligned throughout mechanical loading and rotating towards the *X*-direction under equibiaxial loading. Similar re-orientation and recruitment of collagen fibers were previously demonstrated by Cebral et al.^28^ using multi-photon imaging of aneurysm tissues subjected to uniaxial loading. Additional information regarding the regional analysis of the load-dependent changes in the collagen fiber architecture can be found in Table 2.

**Table 2:** Regional analysis of the quantified collagen fiber orientation angle $\theta _{\mathrm{fiber}}$ and the degree of optical anisotropy (DOA), reported as the median and interquartile range (IQR) for Loading Point 1 and the corresponding percent changes associated with Loading Points 2–4 (see Fig. S1 for the definition of the $3\times 3$ sub-regions).

	$\theta _{\mathrm{fiber}}$		DOA		$\theta _{\mathrm{fiber}}$		DOA	
	Median	0.5*IQR	Median	0.5*IQR	Median	0.5*IQR	Median	0.5*IQR
	Region 1				Region 2			
Loading Point 1	$71.0\,^{\circ }$	$21.3\,^{\circ }$	0.075	0.009	$87.6\,^{\circ }$	$12.4\,^{\circ }$	0.058	0.010
Loading Point 2 vs 1	$-36.8\%$	$-68.3\%$	$+5.1\%$	$+38.8\%$	$-31.5\%$	$-20.7\%$	$+0.7\%$	$-33.5\%$
Loading Point 3 vs 1	$-34.2\%$	$-64.8\%$	$+9.2\%$	$+50.2\%$	$-30.6\%$	$-10.7\%$	$-0.4\%$	$-36.4\%$
Loading Point 4 vs 1	$-35.1\%$	$-59.9\%$	$+9.2\%$	$+52.8\%$	$-33.3\%$	$-19.3\%$	$+6.1\%$	$-28.7\%$
	Region 3				Region 4			
Loading Point 1	$92.2\,^{\circ }$	$10.8\,^{\circ }$	0.057	0.009	$67.1\,^{\circ }$	$16.4\,^{\circ }$	0.082	0.009
Loading Point 2 vs 1	$-5.3\%$	$+31.6\%$	$-5.9\%$	$+36.9\%$	$-16.1\%$	$-52.7\%$	$+10.6\%$	$+19.9\%$
Loading Point 3 vs 1	$-15.0\%$	$+56.3\%$	$-2.1\%$	$+32.3\%$	$-15.4\%$	$+13.4\%$	$-8.7\%$	$+110.7\%$
Loading Point 4 vs 1	$-19.8\%$	$+46.5\%$	$+1.2\%$	$+22.1\%$	$-17.0\%$	$+18.9\%$	$-12.8\%$	$+113.7\%$
	Region 5				Region 6			
Loading Point 1	$90.4\,^{\circ }$	$9.4\,^{\circ }$	0.072	0.009	$63.5\,^{\circ }$	$11.0\,^{\circ }$	0.062	0.008
Loading Point 2 vs 1	$-12.9\%$	$+86.5\%$	$+5.3\%$	$+61.8\%$	$+5.0\%$	$+197.3\%$	$+2.3\%$	$+13.4\%$
Loading Point 3 vs 1	$-0.6\%$	$+0.5\%$	$+1.9\%$	$+34.4\%$	$+19.6\%$	$+91.6\%$	$+9.4\%$	$+19.8\%$
Loading Point 4 vs 1	$-8.3\%$	$+6.4\%$	$+11.0\%$	$+48.0\%$	$+19.5\%$	$+54.1\%$	$+16.2\%$	$+8.9\%$
	Region 7				Region 8			
Loading Point 1	$72.2\,^{\circ }$	$11.0\,^{\circ }$	0.083	0.008	$81.4\,^{\circ }$	$18.3\,^{\circ }$	0.068	0.012
Loading Point 2 vs 1	$+8.9\%$	$+24.4\%$	$-18.9\%$	$+41.8\%$	$-3.6\%$	$-24.5\%$	$-19.3\%$	$-33.7\%$
Loading Point 3 vs 1	$+28.6\%$	$-32.0\%$	$-20.2\%$	$+51.9\%$	$+3.9\%$	$-18.9\%$	$+6.8\%$	$+2.9\%$
Loading Point 4 vs 1	$+27.0\%$	$-31.5\%$	$-14.6\%$	$+65.9\%$	$+1.7\%$	$-36.2\%$	$+9.5\%$	$-6.0\%$
	Region 9							
Loading Point 1	$77.7\,^{\circ }$	$9.5\,^{\circ }$	0.059	0.007				
Loading Point 2 vs 1	$+7.2\%$	$+138.7\%$	$+3.5\%$	$+16.0\%$				
Loading Point 3 vs 1	$-7.6\%$	$-15.0\%$	$+57.7\%$	$+210.2\%$				
Loading Point 4 vs 1	$-10.6\%$	$-40.5\%$	$+92.9\%$	$+214.5\%$				

#### Relating collagen fiber architecture to tissue-level mechanical responses

The observed macroscopic collagen fiber architecture of our human aneurysm tissue can relate to the tissue-level biaxial mechanical testing results. Specifically, the above-mentioned load-dependent changes in the collagen fiber architecture of the aneurysm tissue agree with the smaller stress reduction (Fig. 2d) and more influence on the directional coupling in the *Y*-direction (Fig. 4b), both indicating more non-viscoelastic components in this tissue direction. In contrast, the biaxial tension results displayed a stiffer mechanical response in the *X*-direction compared to the *Y*-direction (Fig. 4a), suggesting a better-aligned collagen fiber network in the *X*-direction. One possible explanation is that although the preferred collagen fiber direction was in the *Y*-direction, the collagen fibers have a relatively large recruitment stretch, i.e., the stretch required for the fibers to become completely uncrimped and straightened to contribute to the tissue’s load bearing capacity. This is supported by the long low-stress toe region (Fig. 4a). The results from prior studies for the aortic valve cusps showed that the tissue mechanical response became stiffer with less fiber crimp^46,47^.

The quantified preferred collagen fiber orientation near the tested *Y*-direction suggests that the tested *Y*-direction may have been close to the arterial circumferential direction. Prior investigations have shown that the collagen fiber architecture preferentially aligns near to the circumferential direction, likely to support the arterial vessel, as it dilates and contracts in response to the pulsatile blood flow. This further supports that the tested *X*-direction and the tested *Y*-direction were approximately aligned near the *in vivo* meridional and circumferential directions, respectively.

### Microstructural morphology

#### From histomorphological analysis

**Figure 5:**
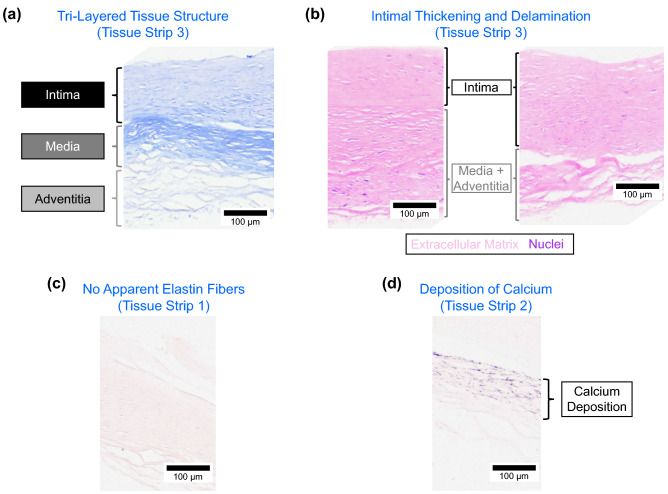
Micrographs of the histology slides showing: (**a**) the three arterial layers (Masson’s trichrome) according to definition in Robertson et al.^20^, (**b**) the thickening of the intimal layer and heterogeneity of the cells (Hematoxylin and Eosin), (**c**) lack of the elastin (Verhoeff van-Gieson), and (**d**) calcium deposition (von Kossa).

Four distinct changes in the aneurysm microstructure were found from visual analyses of the histology slides. First, the Masson’s trichrome stain (Fig. 5a) illustrated the tri-layered structure^20^ and showed that the collagenous media was not significantly altered despite some minor delamination from the intima. However, the adventitia had noticeable circular regions with no stained tissue, indicating the possible deposition of adipose (fat) cells. Secondly, the media and adventitia became difficult to be differentiated for some regions of the tissue, while the intima became delaminated and had large variations in its relative thickness (Fig. 5b). Thirdly, the Verhoeff van-Gieson stain showed that there was very little (if any) elastin in the tissue, suggesting the depletion of the elastin component for the aneurysm tissue (Fig. 5c). Finally, some regions of the aneurysm tissue had layers of calcium deposition, as indicated by the von Kossa stain (Fig. 5d). The histology micrographs for the five tissue strips together with the five stains can be found in the Online Supplementary Information (Fig. S3).

#### From optical coherence tomography (OCT)

**Figure 6:**
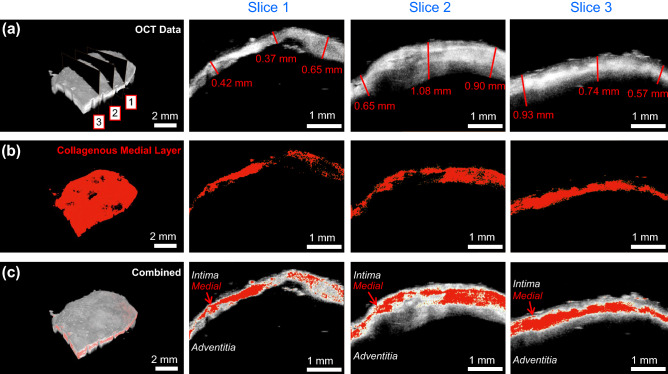
(**a**) Optical coherence tomography (OCT) results for the aneurysm tissue that was analyzed to quantify the (**b**) collagenous medial layer. (**c**) OCT results with the collagenous medial layer highlighted in red.

Volume rendering of the OCT results showed a complex 3D tissue structure that was further analyzed considering three evenly spaced 2D cross sections (Fig. 6a). Quantitative analysis using ImageJ showed that the intra-slice thickness variation is considerable with no discernable trend in the thickness for the selected three slice views (Fig. 6a). For example, the right portion of Slice 1 is thicker (0.65 mm vs 0.37 mm and 0.42 mm), whereas the central portion of Slice 2 is thicker (1.08 mm vs 0.65 mm and 0.90 mm). Similarly, the inter-slice thickness varied noticeably with Slice 2 having the largest thickness (0.65–1.08 mm vs 0.37–0.65 mm and 0.57–0.93 mm).

Further post-processing of the OCT data yielded 3D structural information of the collagenous medial layer (shown as red in Fig. 6b) and a clear 3-layered architecture (Fig. 6c) that agreed with the histological assessment (Fig. 5a). Focusing on the collagenous medial layer, there was a noticeable localized microstructural damage shown in the complete 3D rendering and the 2D cross sections (Fig. 6b) that were not visible in the original OCT data (Fig. 6a). The loss of the demarcation between the tissue layers and destruction of the collagenous media is consistent with the previous observation^48^ and may be correlated to the potential rupture of the aneurysm in vivo. Interestingly, when the medial layer was highlighted in the combined OCT data (Fig. 6c), the adventitia layer appeared to become thicker near the damage to the medial layer. The apparent adaptation of the tissue microstructure to the localized damage may be connected to the cell-mediated growth and remodeling of the aneurysm tissue^49,50^.

#### Comparisons with existing literature

Previously, Frösen et al.^26^ found that the aneurysm tissue progressed to possess a thicker intima with the infiltration of leukocytes. Our results suggested that the intimal thickening varies regionally, even within one histology tissue strip (Fig. 5b), which may have implications on the rupture-potential with localized areas of a higher aneurysm rupture risk. Moreover, we also found that the elastin is completely missing from the tissue in some regions (Fig. 5c). Similar observations were made by Hasegawa et al.^27^ for the aneurysm tissues occluded by the clip blade treatment. Since the elastin is the primary elastic component of the tissue that restores its diastolic configuration, the complete lack of this constituent may indicate one reason for the continuous growth of the aneurysm to its larger size. Furthermore, not only was there obvious damage found in the aneurysm tissue (Fig. 5a,b), but the von Kossa staining also highlighted regions of layer-deposited calcium (Fig. 5d). Although its contribution is not quantifiable, the deposition of calcium may play a role in the unique mechanical behavior of the tissue, such as the directional coupling and seemingly backward anisotropy.

On the other hand, our OCT results provided insight into the regionally varying thickness and the localized tissue microstructural damage and growth and remodeling. Specifically, the 2D cross sections showed a non-uniform thickness of the aneurysm tissue (Fig. 6a) that may indicate the local changes in the aneurysm wall due to the wall shear stress. Meng et al.^51^ previously suggested that the degree of wall shear stress in the aneurysm may be linked to the type of tissue remodeling: low wall shear stress results in small, thin-walled aneurysms whereas high wall shear stress leads to large, thick-walled aneurysms. The aneurysm presented in this study was relatively large with localized non-uniformities in thickness, implying generally large but heterogenous wall shear stresses acting on the aneurysm tissue. Furthermore, our OCT image analysis allowed visualization of the collagenous medial layer (Fig. 6b) that agreed with our histological evaluation (Fig. 5a). In particular, we noted local areas of damage to the collagenous medial layer, in which the demarcation between layers was not clear or the medial layer was completely missing. Interestingly, these local areas of damage to the medial layer corresponded with a thicker adventitia layer, providing another indicator of localized cell-mediated growth and remodeling.

### Clinical implications

The results of this pilot study provided new insight into the function-structure relationship of a human aneurysm tissue, but the specimen number ($n=1$) is limited so the results are not generalizable yet to the clinical setting. Nevertheless, we expect the extensions utilizing our comprehensive characterization framework will provide a significant value to the clinical understanding of aneurysm initiation, growth, and rupture potential. For example, the methods can be used to characterize multiple human aneurysm tissues to better understand the function-structure relationship within the context of a larger sample that has more clinical relevance. Additionally, aneurysm tissues from other cerebral arteries and with different sizes can be characterized to further explore potential region-based and sized-based differences.

The mechanical and structural database from these future characterizations can be used to inform constitutive models that capture the complex mechanical^31,38,52^ and growth-related^49,50^ behaviors of the aneurysm tissue. Then, the models can be implemented into finite element simulations of the aneurysm function and the hemodynamics of the surrounding brain artery environment. Moreover, the developed simulations may be of particular importance when performing risk assessment on smaller aneurysms ($<5\hbox {mm}$). Previously, smaller aneurysms were shown to account for 26% of ruptured aneurysms in a 280-patient cohort with consecutive patient follow-ups of 17 years^53^. In contrast, another clinical study has shown a relatively small annual risk of rupture (0.54%) for smaller aneurysms^54^. Computational modeling tools could provide more insight into this seemingly challenging and debatable aneurysm condition, assist in the decision for clinical intervention, and could be used to evaluate other rupture-related metrics (e.g., the aneurysm-to-vessel size ratio^55^). On the other hand, the developed simulation tools can act as a test bed for newly developed therapeutic options, such as shape memory polymer embolic devices^56,57^ or blood diverting stents^58–60^. Specifically, the fluid-structure interaction type of numerical simulations can be used to evaluate the altered hemodynamic characteristics and how the medical device interacts with the arterial and aneurysm tissues.

### Study limitations and future extensions

There are several limitations in the present study. Firstly, the exact *in vivo* orientation of the resected tissue was unknown, so the information from the present study cannot be fully understood within the context of the *in vivo* function. Regardless, we have successfully quantified the mechanical properties of the tissue and related it to the collagen fiber architecture acquired from the pSFDI imaging. With similar future characterizations of other resected aneurysm tissues, we could compare the collagen fiber architecture to re-address the orientation of the tissue in the present study. This would also allow an expansion of the limited sample size ($n=1$) in the present investigation, which restricts to gain a higher impact of our multi-scale characterization framework. Secondly, the mechanical stretch was computed based on the tine displacement, and a previous study illustrated that optical-based strain calculations more accurately capture the tissue strains^61^. Thirdly, when mounting the tissue to the BioTester, potential misalignment of the tines could result in shear deformations of the tissue that could not be accounted for in the tine-based deformation calculations. Fourthly, we only used the stress relaxation testing (i.e., constant displacement) to understand the viscoelastic properties of the tissue, and the creep testing modality (i.e., constant applied force) may provide additional insight. Fifthly, the load-dependent collagen fiber architecture was only quantified at four points in an equibiaxial loading scenario. However, our biaxial mechanical characterization results showed a unique directional coupling, suggesting that future extensions should explore the non-equibiaxial load-dependent collagen fiber architecture. Finally, the resected aneurysm tissue was temporarily stored in a freezer after surgical removal, and the effect of freezing on aneurysm tissues is not completely understood. Previous studies for other soft tissues found negligible differences before and after temporary freezing^62–64^, but future extensions would ideally use the fresh resected tissue for the characterizations.

This pilot study has presented a multiscale characterization, *the first of its kind*, for aneurysm tissues including mechanical, collagen microstructural, and morphological characterizations. The extensions of this work include both experimental and computational topics. As for experimental extensions, the presented methods could be extended to consider other aneurysms from different locations in the cerebral vasculature and expand on the limited sample size ($n=1$) of the present investigation. Additionally, comprehensive characterizations of ruptured and unruptured aneurysms may provide insight into the role of localized microstructural damage (e.g., the altered collagen fiber architecture) in aneurysm rupture^65^. Ideally, these future extensions would include a healthy arterial control for comparisons, but this is difficult since cerebral arterial tissues would need to be acquired post-mortem. On the other hand, we have performed preliminary constitutive modeling using three common constitutive models. Future extensions should explore microstructurally informed constitutive models^52,66^ that incorporate the aneurysm collagen microstructure, morphology, and tissue-level mechanics to accurately describe the tissue’s mechanical response. Eventually, these constitutive models could be incorporated into computational models of the aneurysm fluid-structure interaction^67–69^ to better understand the growth process and rupture potential to better inform clinical decisions and treatment options.

## Conclusion

This novel multiscale characterization of a resected human aneurysm tissue has provided new insights into the tissue’s mechanical, collagen microstructural, and morphological properties. The biaxial mechanical characterizations of the aneurysm tissue have shown differences in the loading/unloading curves and that the tissue was nonlinear, anisotropic, and had unique directional coupling. The biaxial mechanical data was then fit with three common constitutive models, but future investigations are necessary to explore the use of microstructurally informed constitutive models that can leverage all the mechanical, microstructural, and morphological data. Moreover, the results of the stress relaxation testing have indicated that the aneurysm tissue stress relaxation was more prominent in the *X*-direction, suggesting that there may be more non-viscoelastic components aligned close to the *Y*-direction. Furthermore, collagen microstructural characterizations have indicated that the collagen fibers of the aneurysm tissue were predominantly aligned near the testing *Y*-direction at the unloaded state. As the tissue was gradually loaded under equibiaxial loading, the collagen microstructure slightly rotated away from the *Y*-direction and became more aligned, illustrating the load-dependent properties of the collagen fiber architecture. Finally, in the histomorphological analysis, we have also found that there were significant changes to microstructure of the tissue, including potential adipose cell deposition in the adventitia, thickening of the intima, a lack of elastin, and deposition of calcium near the media/intima interface. These morphological features were supplemented by the OCT analysis that showed a non-uniform tissue thickness and localized damage to the collagenous medial layer. The comprehensive framework presented in this pilot aneurysm tissue characterization study can be extended to other aneurysm tissues from different cerebral arteries and ruptured/unruptured states to further understand the mechanics-microstructure interrelationship. The expanded mechanical and microstructural database could then be used to develop simulation tools for evaluating novel therapeutic options and enhancing the clinical understanding of aneurysm rupture-related risk.

## Supplementary Information

Supplementary Information

## References

[1] Farnham JM (2004). Confirmation of chromosome 7q11 locus for predisposition to intracranial aneurysm. Hum. Genet..

[2] Meng H (2007). Complex hemodynamics at the apex of an arterial bifurcation induces vascular remodeling resembling cerebral aneurysm initiation. Stroke.

[3] Hosaka K, Hoh BL (2014). Inflammation and cerebral aneurysms. Transl. Stroke Res..

[4] Investigators UJ (2012). The natural course of unruptured cerebral aneurysms in a Japanese cohort. N. Engl. J. Med..

[5] Kouskouras C (2004). Intracranial aneurysms: Evaluation using CTA and MRA. Correlation with DSA and intraoperative findings. Neuroradiology.

[6] van Rooij WJ, Sprengers ME, de Gast AN, Peluso JPP, Sluzewski M (2008). 3D rotational angiography: The new gold standard in the detection of additional intracranial aneurysms. Am. J. Neuroradiol..

[7] Jayaraman MV (2004). Detection of intracranial aneurysms: Multi-detector row CT angiography compared with DSA. Radiology.

[8] Burrel M (2003). MRI angiography is superior to helical CT for detection of HCC prior to liver transplantation: An explant correlation. Hepatology.

[9] Lu J, Liu J-C, Wang L-J, Qi P, Wang D-M (2012). Tiny intracranial aneurysms: Endovascular treatment by coil embolisation or sole stent deployment. Eur. J. Radiol..

[10] Niimi Y, Song J, Madrid M, Berenstein A (2006). Endosaccular treatment of intracranial aneurysms using matrix coils: Early experience and midterm follow-up. Stroke.

[11] Raymond J (2003). Long-term angiographic recurrences after selective endovascular treatment of aneurysms with detachable coils. Stroke.

[12] Regli L, Uske A, de Tribolet N (1999). Endovascular coil placement compared with surgical clipping for the treatment of unruptured middle cerebral artery aneurysms: A consecutive series. J. Neurosurg..

[13] Claiborne, J. S. *et al.* Endovascular and surgical treatment of unruptured cerebral aneurysms: Comparison of risks. *Ann. Neurol.* **48**, 11–19. 10.1002/1531-8249(200007)48:1$$<$$11::aid-ana4$$>$$3.3.co;2-m (2000). 10.1002/1531-8249(200007)48:1<11::aid-ana4>3.3.co;2-m 10894211

[14] Higashida RT (2007). Treatment of unruptured intracranial aneurysms: A nationwide assessment of effectiveness. American Journal of Neuroradiology.

[15] Brunel H (2018). Rupture limit evaluation of human cerebral aneurysms wall: Experimental study. J. Biomech..

[16] Costalat V (2011). Biomechanical wall properties of human intracranial aneurysms resected following surgical clipping (IRRAs Project). J. Biomech..

[17] Lipovka AI, Ovsyannikov KS, Dubovoy AV, Parshin DV (2018). On the mechanics of a fusiform cerebral aneurysm: Mooney-Rivlin mathematical model for the experimental data. J. Phys: Conf. Ser..

[18] Parshin DV, Kuianova IO, Yunoshev AS, Ovsyannikov KS, Dubovoy AV (2017). On the mechanics of cerebral aneurysms: Experimental research and numerical simulation. J. Phys: Conf. Ser..

[19] Parshin D (2019). Different stages of the evolution of cerebral aneurysms: Joint analysis of mechanical test data and histological analysis of aneurysm tissue. EPJ Web of Conferences.

[20] Robertson AM (2015). Diversity in the strength and structure of unruptured cerebral aneurysms. Ann. Biomed. Eng..

[21] Scott S, Ferguson GG, Roach MR (1972). Comparison of the elastic properties of human intracranial arteries and aneurysms. Can. J. Physiol. Pharmacol..

[22] Seshaiyer P, Hsu FPK, Shah AD, Kyriacou SK, Humphrey JD (2001). Multiaxial mechanical behavior of human saccular aneurysms. Comput. Methods Biomech. Biomed. Eng..

[23] Steiger HJ, Aaslid R, Keller S, Reulen H-J (1989). Strength, elasticity and viscoelastic properties of cerebral aneurysms. Heart Vessels.

[24] Tóth BK, Raffai G, Bojtár I (2005). Analysis of the mechanical parameters of human brain aneurysm. Acta Bioeng. Biomech..

[25] Valencia A (2015). Mechanical test of human cerebral aneurysm specimens obtained from surgical clipping. J. Mech. Med. Biol..

[26] Frösen J (2004). Remodeling of saccular cerebral artery aneurysm wall is associated with rupture: Histological analysis of 24 unruptured and 42 ruptured cases. Stroke.

[27] Hasegawa T, Horiuchi T, Hongo K (2015). Histological analysis of aneurysm wall occluded with clip blades. A case report. World Neurosurg..

[28] Cebral JR (2015). Wall mechanical properties and hemodynamics of unruptured intracranial aneurysms. Am. J. Neuroradiol..

[29] Signorelli F (2018). Biomechanical characterization of intracranial aneurysm wall: A multiscale study. World Neurosurg..

[30] Tong P, Fung Y-C (1976). The stress-strain relationship for the skin. J. Biomech..

[31] Holzapfel GA, Gasser TC, Ogden RW (2000). A new constitutive framework for arterial wall mechanics and a comparative study of material models. J. Elast..

[32] Rodriguez JF, Martufi G, Doblaré M, Finol EA (2009). The effect of material model formulation in the stress analysis of abdominal aortic aneurysms. Ann. Biomed. Eng..

[33] Gasser TC, Ogden RW, Holzapfel GA (2005). Hyperelastic modelling of arterial layers with distributed collagen fibre orientations. J. R. Soc. Interface.

[34] Yu W-J (2014). Differential evolution with two-level parameter adaptation. IEEE Trans. Cybern..

[35] Jett SV (2020). Integration of polarized spatial frequency domain imaging (pSFDI) with a biaxial mechanical testing system for quantification of load-dependent collagen architecture in soft collagenous tissues. Acta Biomater..

[36] Tang Q (2016). Depth-resolved imaging of colon tumor using optical coherence tomography and fluorescence laminar optical tomography. Biomed. Opt. Express.

[37] Wang Y, Wang J, Song X, Han L (2016). An efficient adaptive fuzzy switching weighted mean filter for salt-and-pepper noise removal. IEEE Signal Process. Lett..

[38] Zhang P, Li F (2014). A new adaptive weighted mean filter for removing salt-and-pepper noise. IEEE Signal Process. Lett..

[39] Molina I, Martinez E, Arquero A, Pajares G, Sanchez J (2012). Evaluation of a change detection methodology by means of binary thresholding algorithms and informational fusion processes. Sensors.

[40] Lanir Y, Fung YC (1974). Two-dimensional mechanical properties of rabbit skin-II. Experimental results. J. Biomech..

[41] Jett SV (2018). An investigation of the anisotropic mechanical properties and anatomical structure of porcine atrioventricular heart valves. J. Mech. Behav. Biomed. Mater..

[42] Dobrin PB (1978). Mechanical properties of arteries. Physiol. Rev..

[43] May-Newman K, Yin FC (1995). Biaxial mechanical behavior of excised porcine mitral valve leaflets. Am. J. Physiol. Heart Circ. Physiol..

[44] Liao J, Vesely I (2004). Relationship between collagen fibrils, glycosaminoglycans, and stress relaxation in mitral valve chordae tendineae. Ann. Biomed. Eng..

[45] Elliott DM (2003). Effect of altered matrix proteins on quasilinear viscoelastic properties in transgenic mouse tail tendons. Ann. Biomed. Eng..

[46] Sacks MS, Smith DB, Hiester ED (1997). A small angle light scattering device for planar connective microstructural analysis. Ann. Biomed. Eng..

[47] Billiar KL, Sacks MS (2000). Biaxial mechanical properties of the natural and glutaraldehyde treated aortic valve cusp-Part I: Experimental result. J. Biomech. Eng..

[48] Liu Y, Zheng Y, An Q, Song Y, Leng B (2019). Optical coherence tomography for intracranial aneurysms: A new method for assessing the aneurysm structure. World Neurosurg..

[49] Cyron CJ, Humphrey JD (2017). Growth and remodeling of load-bearing biological soft tissues. Meccanica.

[50] Kroon M, Holzapfel GA (2009). A theoretical model for fibroblast-controlled growth of saccular cerebral aneurysms. J. Theor. Biol..

[51] Meng H, Tutino VM, Xiang J, Siddiqui A (2014). High WSS or low WSS? complex interactions of hemodynamics with intracranial aneurysm initiation, growth, and rupture: Toward a unifying hypothesis. Am. J. Neuroradiol..

[52] Holzapfel GA, Niestrawska JA, Ogden RW, Reinisch AJ, Schriefl AJ (2015). Modelling non-symmetric collagen fibre dispersion in arterial walls. J. R. Soc. Interface.

[53] Ohashi Y, Horikoshi T, Sugita M, Yagishita T, Nukui H (2004). Size of cerebral aneurysms and related factors in patients with subarachnoid hemorrhage. Surg. Neurol..

[54] Sonobe M, Yamazaki T, Yonekura M, Kikuchi H (2010). Small unruptured intracranial aneurysm verification study: SUAVe study. Japan. Stroke.

[55] Kashiwazaki D, Kuroda S (2013). Size ratio can highly predict rupture risk in intracranial small ($$<5 \text{mm}$$) aneurysms. Stroke.

[56] Maitland DJ (2007). Prototype laser-activated shape memory polymer foam device for embolic treatment of aneurysms. J. Biomed. Opt..

[57] Wang J (2019). Shape memory polyurethane with porous architectures for potential applications in intracranial aneurysm treatment. Polymers.

[58] Cebral JR (2011). Aneurysm rupture following treatment with flow-diverting stents: Computational hemodynamics analysis of treatment. Am. J. Neuroradiol..

[59] Chiu AHY, Wenderoth J (2013). Cerebral hyperperfusion after flow diversion of large intracranial aneurysms. J. Neurointervent. Surg..

[60] Levitt MR (2014). Cerebral aneurysms treated with flow-diverting stents: Computational models with intravascular blood flow measurements. Am. J. Neuroradiol..

[61] Sacks MS (2000). Biaxial mechanical evaluation of planar biological materials. J. Elastic. Phys. Sci. Solids.

[62] Clavert P (2001). Effects of freezing/thawing on the biomechanical properties of human tendons. Surg. Radiol. Anat..

[63] Delgadillo JOV, Delorme S, El-Ayoubi R, DiRaddo R, Hatzikiriakos SG (2010). Effect of freezing on the passive mechanical properties of arterial samples. J. Biomed. Sci. Eng..

[64] Duginski GA, Ross CJ, Laurence DW, Johns CH, Lee C-H (2020). An investigation of the effect of freezing storage on the biaxial mechanical properties of excised porcine tricuspid valve anterior leaflets. J. Mech. Behav. Biomed. Mater..

[65] Eriksson T, Kroon M, Holzapfel GA (2009). Influence of medial collagen organization and axial in situ stretch on saccular cerebral aneurysm growth. J. Biomech. Eng..

[66] Zhang W, Ayoub S, Liao J, Sacks MS (2016). A meso-scale layer-specific structural constitutive model of the mitral heart valve leaflets. Acta Biomater..

[67] Torii R, Oshima M, Kobayashi T, Takagi K, Tezduyar TE (2008). Fluid-structure interaction modeling of a patient-specific cerebral aneurysm: Influence of structural modeling. Comput. Mech..

[68] Torii R, Oshima M, Kobayashi T, Takagi K, Tezduyar TE (2009). Fluid-structure interaction modeling of blood flow and cerebral aneurysm: Significance of artery and aneurysm shapes. Comput. Methods Appl. Mech. Eng..

[69] Watton PN (2011). Modelling evolution and the evolving mechanical environment of saccular cerebral aneurysms. Biomech. Model. Mechanobiol..

