# Supplementary material for: A pilot study on biaxial mechanical, collagen microstructural, and morphological characterizations of a resected human intracranial aneurysm tissue
Source: Sci Rep. 2021 Feb 10;11:3525. doi: 10.1038/s41598-021-82991-x (PMC7876029; doi:10.1038/s41598-021-82991-x)
Supplement: Supplementary file 1 — Supplementary Information [file 41598_2021_82991_MOESM1_ESM.pdf]

Supplementary Information

This Supplementary Information section provides the supplemental information for the manuscript "*A pilot study on biaxial mechanical, collagen microstructural, and morphological characterizations of a resected human intracranial aneurysm tissue*" authored by Devin W. Laurence, Hannah Homburg, Feng Yan, Qinggong Tang, Kar-Ming Fung, Bradley N. Bohnstedt, Gerhard A. Holzapfel, and Chung-Hao Lee. Specifically, **Tables S1-S3** provide the model parameters from the 10 optimization replicates for each of the chosen three constitutive models, **Figure S1,S2** provide the region-based analysis of the polarized spatial frequency domain imaging (pSFDI) results, and **Figure S3** provides the comprehensive histological information for the human aneurysm tissue.

Table S1 – Summary of the constitutive model parameters for the 10 replicates using the Fung-type model (Eq. 1).

Replicate	Parameter				R^2
	c (kPa)	c_1 (-)	c_2 (-)	c_3 (-)	
1	1.915	8.720	2.581	0.436	0.977
2	1.914	8.721	2.581	0.436	0.977
3	1.915	8.720	2.581	0.436	0.977
4	1.916	8.720	2.581	0.434	0.977
5	1.916	8.714	2.580	0.437	0.977
6	1.915	8.720	2.581	0.436	0.977
7	1.915	8.720	2.581	0.436	0.977
8	1.915	8.721	2.581	0.436	0.977
9	1.914	8.721	2.581	0.436	0.977
10	1.915	8.721	2.581	0.436	0.977
Mean	1.915	8.720	2.581	0.436	
SEM	0.0002	0.0007	0.0001	2E-04	

Table S2 – Summary of the constitutive model parameters for the 10 replicates using the Holzapfel-Gasser-Ogden (HGO) model (Eq. 2).

Replicate	Parameter							R^2
	C_{10} (kPa)	k_1 (kPa)	k_2 (-)	k_3 (kPa)	k_4 (-)	θ_1 (deg.)	θ_2 (deg.)	
1	3.069	2.988	1.293	3.569	4.699	59.4	30.2	0.987
2	3.066	2.988	1.292	3.572	4.697	59.5	30.2	0.988
3	2.359	3.436	1.340	4.104	4.527	58.0	29.3	0.988
4	2.921	3.103	1.295	3.617	4.697	59.2	30.0	0.987
5	3.067	2.988	1.292	3.568	4.699	59.5	30.2	0.988
6	5.330	2.229	1.511	1.871	5.554	58.0	30.1	0.986
7	3.073	2.985	1.293	3.569	4.698	59.4	30.2	0.987
8	3.010	2.995	1.297	3.576	4.702	59.4	30.1	0.987
9	3.077	2.968	1.294	3.582	4.693	59.5	30.2	0.987
10	3.067	2.986	1.293	3.572	4.697	59.5	30.2	0.988
Mean	3.067	2.986	1.293	3.572	4.697	59.1	30.0	
SEM	0.246	0.184	0.089	0.093	0.022	0.2	0.1	

Table S3 – Summary of the constitutive model parameters for the 10 replicates using the Gasser-Ogden-Holzapfel (GOH) model (Eq. 3).

Replicate	Parameter							R^2
	C_{10} (kPa)	k_1 (kPa)	k_1 (-)	κ_1 (-)	κ_2 (-)	θ_1 (deg.)	θ_2 (deg.)	
1	4.031	6.702	4.384	0.251	0.031	85.0	28.7	0.984
2	4.015	6.637	4.371	0.251	0.030	85.0	28.8	0.983
3	4.019	6.631	4.371	0.251	0.030	85.0	28.8	0.983
4	3.998	6.622	4.359	0.250	0.029	85.0	28.8	0.985
5	4.015	6.636	4.373	0.251	0.030	85.0	28.8	0.984
6	4.013	6.648	4.373	0.251	0.030	85.0	28.7	0.983
7	4.092	7.117	4.533	0.255	0.041	85.0	27.9	0.984
8	3.687	7.240	4.333	0.252	0.033	84.7	28.2	0.983
9	4.016	6.632	4.370	0.251	0.030	85.0	28.8	0.983
10	4.038	6.629	4.380	0.251	0.030	85.0	28.7	0.984
Mean	3.992	6.750	4.385	0.251	0.032	85.0	28.6	
SEM	0.035	0.072	0.017	0.000	0.001	0.0	0.1	

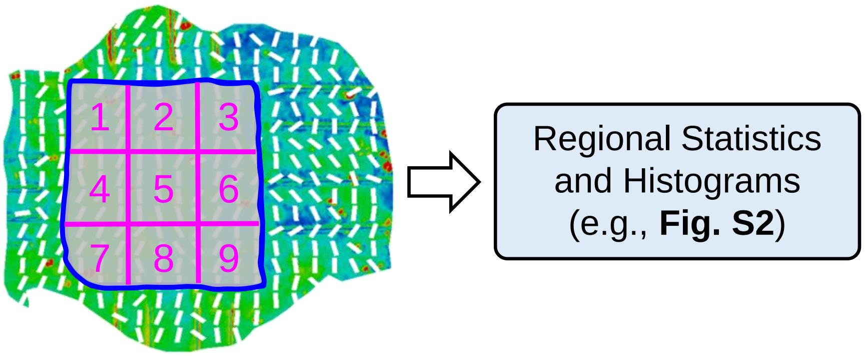

Figure S1 – Tissue's region of interest divided into nine sub-regions, with data from each sub-region used to create the regional histograms (**Fig. S2**) and statistics (**Tables S4,S5**).

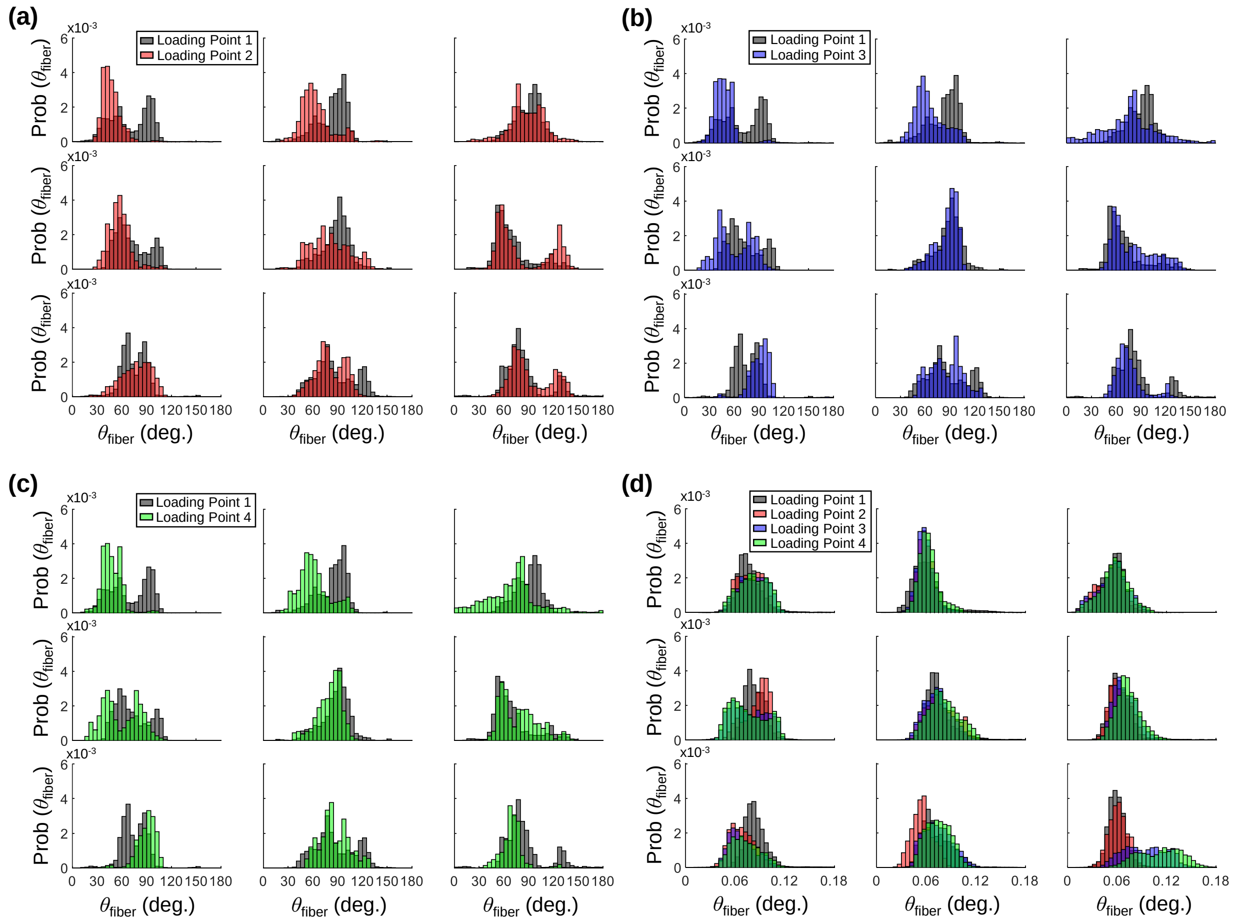

Figure S2 – Regional analysis of the collagen fiber architecture quantified from the pSFDI method for the equibiaxial tension protocol ($F_X : F_Y = 1:1$): comparisons of the preferred fiber orientation of *Loading Point 1* (i.e., the unloaded configuration) with (a) *Loading Point 2*, (b) *Loading Point 3*, and (c) *Loading Point 4*. (d) Comparison of the degree of optical anisotropy (DOA) for all four loading points.

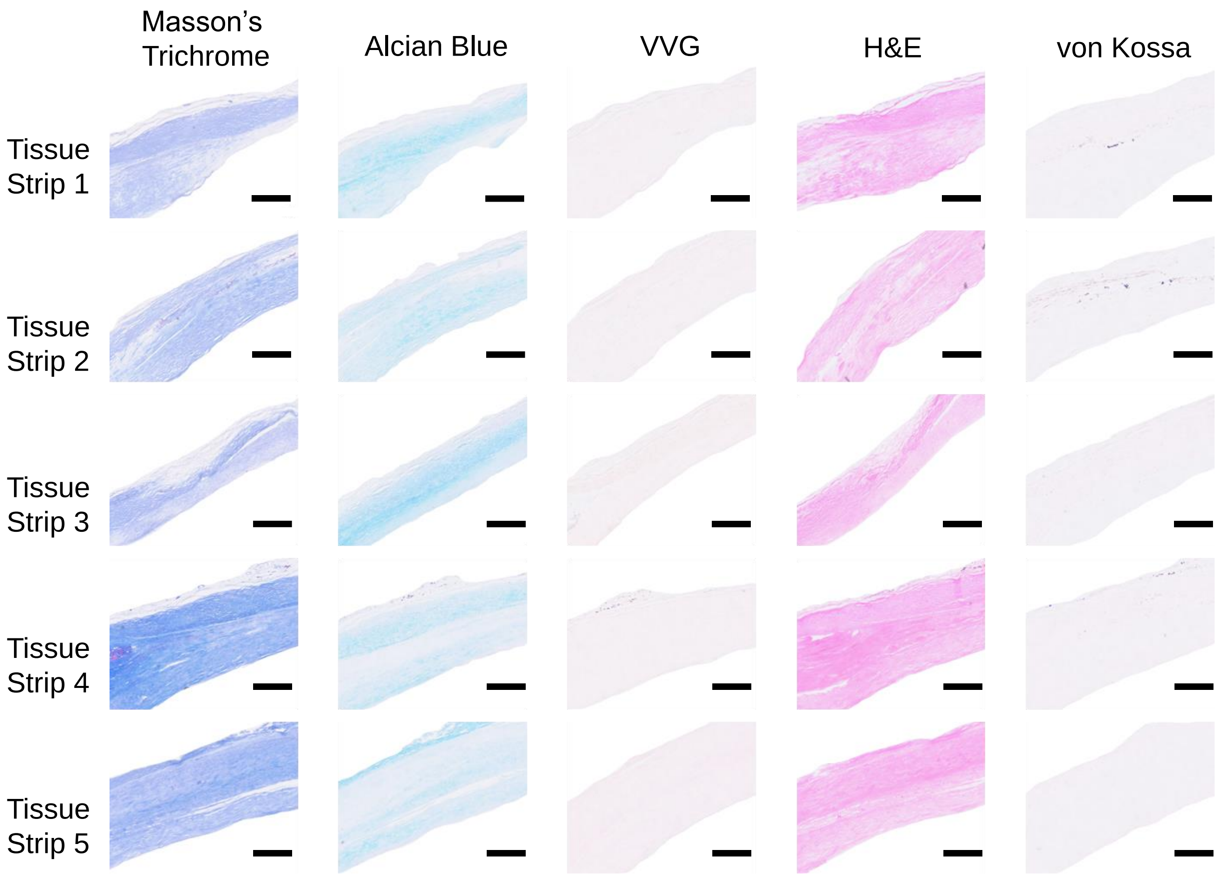

Figure S3 – Histology micrographs for all five histology tissue strips (see the definition in **Fig. 6f**), considering all five stains used in this study. The scale bar denotes 100 µm.
